# Frequency and characteristics of contralateral breast abnormalities following recall at screening mammography

**DOI:** 10.1007/s00330-018-5370-x

**Published:** 2018-04-17

**Authors:** Joost R. C. Lameijer, Angela MP Coolen, Adri C. Voogd, Luc J. Strobbe, Marieke W. J. Louwman, Dick Venderink, Vivian C. Tjan-Heijnen, Lucien E. M. Duijm

**Affiliations:** 1 Department of Radiology, Catharina Hospital, Michelangelolaan 2, 5623EJ Eindhoven, The Netherlands; 2 Department of Radiology, Elisabeth-Tweesteden Hospital (ETZ), Hilvarenbeekseweg 60, 5022 GC Tilburg, The Netherlands; 3 Department of Epidemiology, Maastricht University, P Debyelaan 1, 6229 HA Maastricht, The Netherlands; 4 Department of Research, Netherlands Comprehensive Cancer Organization (IKNL), PO Box 19079, 3501 DB Utrecht, The Netherlands; 5 Department of Internal Medicine, Division of Medical Oncology, GROW Maastricht University Medical Centre, PO Box 5800, 6202 AZ Maastricht, The Netherlands; 6 Department of Radiology, Canisius Wilhelmina Hospital, Weg door Jonkerbos 100, 6532 SZ Nijmegen, The Netherlands; 7 Dutch Expert Centre for Screening, PO Box 6873, 6503 GJ Nijmegen, The Netherlands

**Keywords:** Mammography, Mass screening, Breast neoplasms, Breast, Radiology

## Abstract

**Purpose:**

To determine the frequency and characteristics of contralateral, non-recalled breast abnormalities following recall at screening mammography.

**Methods:**

We included a series of 130,338 screening mammograms performed between 1 January 2014 and 1 January 2016. During the 1-year follow-up, clinical data were collected for all recalls. Screening outcome was determined for recalled women with or without evaluation of contralateral breast abnormalities.

**Results:**

Of 3,995 recalls (recall rate 3.1%), 129 women (3.2%) underwent assessment of a contralateral, non-recalled breast abnormality. Most lesions were detected at clinical mammography and/or breast tomosynthesis (101 women, 78.3%). The biopsy rate was similar for recalled lesions and contralateral, non-recalled lesions, but the positive predictive value of biopsy was higher for recalled lesions (*p* = 0.01). A comparable proportion of the recalled lesions and contralateral, non-recalled lesions were malignant (*p* = 0.1). The proportion of ductal carcinoma in situ was similar for both groups, as well as invasive cancer characteristics and type of surgical treatment.

**Conclusions:**

About 3% of recalled women underwent evaluation of contralateral, non-recalled breast lesions. Evaluation of the contralateral breast after recall is important as we found that 15.5% of contralateral, non-recalled lesions were malignant. Contralateral cancers and screen-detected cancers show similar characteristics, stage and surgical treatment.

**Key Points:**

*• 3% of recalled women underwent evaluation of contralateral, non-recalled lesions*

*• One out of seven contralateral, non-recalled lesions was malignant*

*• A contralateral cancer was diagnosed in 0.5% of recalls*

*• Screen-detected cancers and non-recalled, contralateral cancers showed similar histological characteristics*

*• Tumour stage and surgical treatment were similar for both groups*

## Introduction

Many countries have introduced breast cancer screening programmes with the aim to detect breast cancers at an early stage, before these grow large enough to become symptomatic, and thus to decrease morbidity and improve breast cancer survival. Breast cancer mortality has decreased in the past 20 years in The Netherlands and this improved survival is due to a combination of breast cancer screening and improved treatment [1–3].

Breast cancers detected at screening are often small and non-symptomatic. Improved radiological breast imaging techniques, including the replacement of screen-film mammography by full-field digital mammography (FFDM) and the recent introduction of breast tomosynthesis (three-dimensional mammography), have resulted in a more accurate detection and characterisation of breast lesions at screening mammography [4, 5]. Breast tomosynthesis has not yet been implemented in our nation-wide screening mammography programme. Women are usually recalled for a unilateral lesion at screening mammography, but a small percentage of cases (1-2%) is recalled for suspicious bilateral lesions [6].

After recall, additional breast imaging is performed for the evaluation of the suspected lesion detected at screening mammography. In a diagnostic setting, the addition of breast tomosynthesis to digital mammography results in a higher sensitivity and specificity of breast cancer diagnosis than digital mammography alone [7–9]. For example, Gilbert et al. [9] reported a higher sensitivity for the detection of invasive cancers sized 11-20 mm when adding tomosynthesis to digital mammography in a diagnostic setting (93% versus 86%, *p* < 0.001). Specificity also increased, from 58% with digital mammography to 69% with digital mammography plus breast tomosynthesis. Lång et al. [10] found an increased lesion visualisation with breast tomosynthesis compared to digital mammography, especially for spiculated masses.

Additional breast cancers may be detected in the breast for which the woman has been recalled, or in the contralateral breast. Preoperative breast magnetic resonance imaging (MRI) in breast cancer patients has also been shown to detect additional malignancies in one or both breasts in a significant number of patients, and the MRI findings frequently result in more extensive surgery compared to the initially proposed treatment, such as additional contralateral mastectomy [11–13].

To our knowledge, no previous studies have reported on additionally detected contralateral breast lesions in women recalled for a unilateral abnormality at screening mammography. We, therefore, assessed the frequency and characteristics of these contralateral lesions in women who attended a biennial screening mammogram programme in the South of The Netherlands.

## Materials and methods

### Study design and study population

This is a prospective observational follow-up study of women aged 50-75 who attended a biennial breast cancer screening programme conducted in the south of The Netherlands. A consecutive series of 130,338 full-field digital mammography screens (13,762 initial screens and 116,576 subsequent screens) between 1 January 2014 and 1 January 2016 were included. The screening mammograms were obtained at four specialised screening units in a biennial screening mammography programme conducted in the south of The Netherlands.

Women are personally invited by letter to attend the screening programme. These letters are sent to the address of every woman aged between 50 and 75 years old registered in the municipal registration. Women being treated for breast cancer or those attending clinical follow-up after treatment of breast cancer do not attend the screening programme. Otherwise, there are no exclusion criteria for screening eligibility.

Women participating in our screening mammography programme were asked for permission to use their data for scientific purposes and for the evaluation of the screening programme. All women gave permission to use their screening and diagnostic data.

Possible exclusion criteria included no permission to use screen data, technical failure of screening equipment and insufficient image quality as assessed by an experienced screening radiologist. None of the women screened for study inclusion met these criteria.

A total of 3,995 women were recalled for further analysis. The hospitals involved in these recalls were visited and data as described below were obtained. Possible exclusion criteria after recall included insufficient follow-up or loss to follow-up, incomplete records, no permission to access data. None of the women recalled for analysis met these criteria and no recalls were excluded from analysis.

Ethical approval by our local Institutional Review Board was not required for this prospective observational follow-up study, according to the Dutch Central Committee on Research involving Human Subjects (CCMO).

### Screening procedure and recall

Details of our breast cancer screening programme have been described previously [14]. In brief, the mammography screening programme in The Netherlands is a nationwide programme that provides free biennial screening mammography for women aged 50-75 years. Women are personally invited for screening and the attendance rate is more than 80%. Before each screening examination, the woman completes a short questionnaire with questions about previous recalls and any previous breast surgery or breast malignancy.

All digital mammograms were obtained with a Lord Selenia FFDM system (Hologic, Danbury, CT, USA), with a 70-μm pixel size and a 232 × 286-mm field of view. The examinations were obtained by specialised screening mammography radiographers and all screening mammograms were double-read in a blinded fashion by a team of 12 certified screening radiologists. Each radiologist evaluated at least 3,000 mammograms yearly.

The screening radiologists classified abnormal mammographic findings into one of the following categories: suspicious mass, suspicious calcifications, suspicious mass in combination with calcifications, architectural distortion, asymmetry or other. Each screen was then classified according to the BIRADS lexicon [15]. Women with a BI-RADS 1 or 2 were not recalled and women with a BI-RADS 0, 4 or 5 were referred to a dedicated breast unit of a hospital for further analysis of their mammographic finding. The BI-RADS category 3 is not applied as short-term follow-up is not available in the Dutch screening programme.

### Diagnostic work-up after recall

Although a total of 20 hospitals were involved in the diagnostic work-up of recalled women, the diagnostic work-up in the majority of women (97.2%, 3,883/3,995) was performed in seven regional hospitals. All recalled women underwent physical examination by a surgical oncologist or dedicated breast nurse and received additional evaluation at the radiology department. A radiologist first reassessed the screening mammogram of both breasts, which was routinely available and stored in the Picture Archiving and Communication System (PACS) of the hospital. Additional imaging was obtained at the discretion of the radiologist after this review. Full-field digital mammography was available in each of the seven regional hospitals. Breast tomosynthesis was present in two hospitals from the beginning of the inclusion period and became available in three others in 2015. Breast ultrasonography was used for the further characterisation of mammographic abnormalities and palpable breast lesions, for biopsy guidance and for target or second look purposes following breast MRI. Whole-breast ultrasonography was not encouraged, in accordance with the Dutch guidelines [16]. Breast MRI was also available in each of the seven hospitals and performed if indicated, as defined by the guidelines of the European Society of Breast Imaging [17] and the Dutch guidelines. Fine-needle aspiration biopsy (FNAB), core-needle biopsy (CNB) and stereotactic biopsy were performed in each of the seven hospitals, whereas MRI guided biopsy procedures were concentrated in the larger hospitals. All recalls were discussed by multidisciplinary teams that consisted of surgical oncologists, radiologists, medical oncologists, radiation oncologists, plastic surgeons, breast nurses and breast radiographers.

During 1-year follow-up, clinical data and data from diagnostic breast imaging, biopsy specimen and surgical procedures were collected of all recalled women. Information on breast density, hormonal replacement therapy and family history of breast cancer were also extracted from the screening records and clinical data. Breast cancers were categorised into ductal carcinoma in situ (DCIS) and invasive cancers; lobular carcinoma in situ was considered a non-malignant lesion. The TNM (tumour, nodes and metastases) classification was used for malignant lesions [18]. For all cancers treated by neo-adjuvant therapy (either chemotherapy or anti-hormonal therapy), tumour size was derived from breast imaging (usually MRI) prior to the start of this therapy.

### Detection and assessment of contralateral breast lesions

The methods of detection and subsequent assessment of contralateral breast lesions were derived from the clinical records and clinical radiology reports. Similar to the work-up of screen-detected mammographic abnormalities, biopsy data and surgical reports of contralateral breast lesions were obtained.

Two screening radiologists reviewed the latest screening mammogram of each woman with a cancer diagnosed in the contralateral breast. They classified the cancer as missed, minimal sign or occult at the previous screen according to the European guidelines [19, 20].

### Statistical analysis, missing data and bias

Descriptive statistics were performed using Statistical Package for Social Science 23.0 (SPSS IBM, Chicago, IL, USA). The chi-squared test was used to test for differences between women without and with additional contralateral breast lesions detected following recall. A *p* value of less than 0.05 was considered to indicate a statistically significant difference. The *p* values were two sided.

There was no loss to follow-up. In a small number of cases, data were missing (e.g. oestrogen or progesterone receptor status missing due to an insufficient tissue sample). Statistical correction was not performed for the very small numbers of missing data, and data missing was considered to be at random.

Bias was not expected in recall of women who attended screening. In the diagnostic setting, several types of bias may occur, especially observer bias and detection bias, but statistical correction for this type of bias is not feasible in this study. Selection bias was considered highly unlikely as all women analysed in the diagnostic setting were included for statistical analysis.

## Results

### Overall screening results

**Fig. 1:**
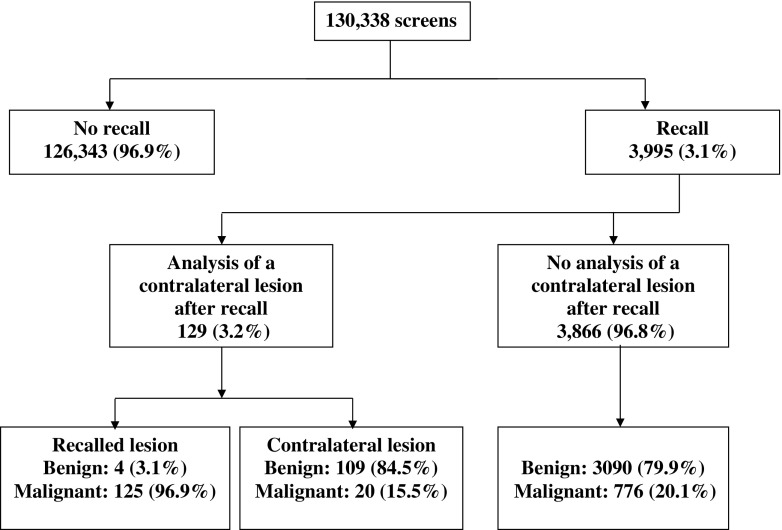
Flow chart of study population

The mean age of the 130,338 consecutively screened women was 59.6 years (95% CI, 59.4-59.8 years) and 8.2% reported to have had previous breast surgery. A total of 3,995 (3.1%) were recalled for further assessment. Breast cancer was diagnosed in 905 recalled women [including 163 (18.0%) ductal carcinoma in situ], resulting in an overall cancer detection rate of 6.9 per 1,000 screens [initial screens, 8.6 (118/13,762); subsequent screens, 6.8 (787/116,576)] and an overall positive predictive value (PPV) of recall of 22.7% [initial screens, 11.4% (118/1,037); subsequent screens, 26.6% (787/2,958)]. A patient flow chart is provided in Fig. 1.

### Detection of contralateral lesions

A total of 129 women, 3.2% of all recalls, underwent assessment of a contralateral breast abnormality that was detected after recall. Baseline characteristics of recalled women, with or without analysis of a contralateral non-recalled lesion, are shown in Table 1. These characteristics were similar for both groups except for a higher breast density (III + IV) in women assessed for a contralateral breast lesion when compared to a recalled breast lesion (38.8% and 23.8% respectively, *p* < 0.001).

**Table 1:** Baseline characteristics of recalled women

	No contralateral lesions *n* = 3,866	Contralateral lesions *n* = 129	*p* value
Mean age, years (95% CI)	59.1 (58.8 – 59.4)	58.4 (57.1–59.8)	0.35
Screening round, No (%)			0.13
Initial Subsequent	996 (25.8) 2,870 (74.2)	41 (31.8) 88 (68.2)	
Breast density, No (%)			<0.001
I + II (<50%) III + IV (>50%)	2,946 (76.2) 920 (23.8)	79 (61.2) 50 (38.8)	
Previous breast surgery, No (%)			0.69
Yes No	322 (8.3) 3,544 (91.7)	12 (9.3) 117 (90.7)	
Hormone replacement therapy, No (%)			0.11
Yes No	159 (4.1) 3,707 (95.9)	9 (7.0) 120 (93.0)	
Family history of breast cancer, No (%)			0.61
Yes No	509 (13.2) 3,357 (86.8)	19 (14.7) 110 (85.3)	

The contralateral abnormality was detected at clinical mammography and/or breast tomosynthesis in 101 women and at breast MRI in 23 women. In the remaining five women, the contralateral breast was evaluated due to the presence of a suspicious finding at clinical breast examination by the surgical oncologist or breast nurse (palpable breast lesion, four cases; Paget disease of the nipple, one case).

The mammographic characteristics of the contralateral lesions detected at clinical mammography (with/without tomosynthesis) were as follows: suspicious mass, 90; suspicious calcifications, 8; suspicious mass with calcifications, 1; architectural distortion, 2. The indications for breast MRI in the 23 women with a contralateral lesion detected at MRI had been as follows: problem solving of the recalled lesion, 9 women; determination of tumour size and/or multifocality/multicentricity of a screen detected malignancy, 10 women; invasive lobular cancer, 4 women. Breast MRI showed a suspicious mass or non-mass enhancement in 19 women and 4 women, respectively.

### Work-up of lesions detected in the contralateral breast

The majority of women with an abnormality detected in the contralateral breast underwent only breast imaging for the further evaluation of these lesions (Table 2). A total of 74 women (57.4%) received breast ultrasonography and the remaining 55 women (42.6%) underwent one or several percutaneous and/or excisional biopsy procedures to establish a final pathological diagnosis.

**Table 2:** Work-up of lesions detected in the contralateral breast during recall

Breast ultrasonography, No (%)	74 (57.4)	
Breast ultrasonography plus biopsy, No (%)		
FNAC CNB SCNB FNAC + CNB CNB + SCNB Percutaneous biopsy + excisional biopsy Excisional biopsy	1 (0.8) 36 (27.9) 10 (7.8) 1 (0.8) 2 (1.6) 4 (3.1) 1 (0.8)	

*FNAC* fine-needle aspiration cytology, *CNB* core-needle biopsy, *SCNB* stereotactic core-needle biopsy

### Outcome of lesions detected in the contralateral breast

**Fig. 2:**
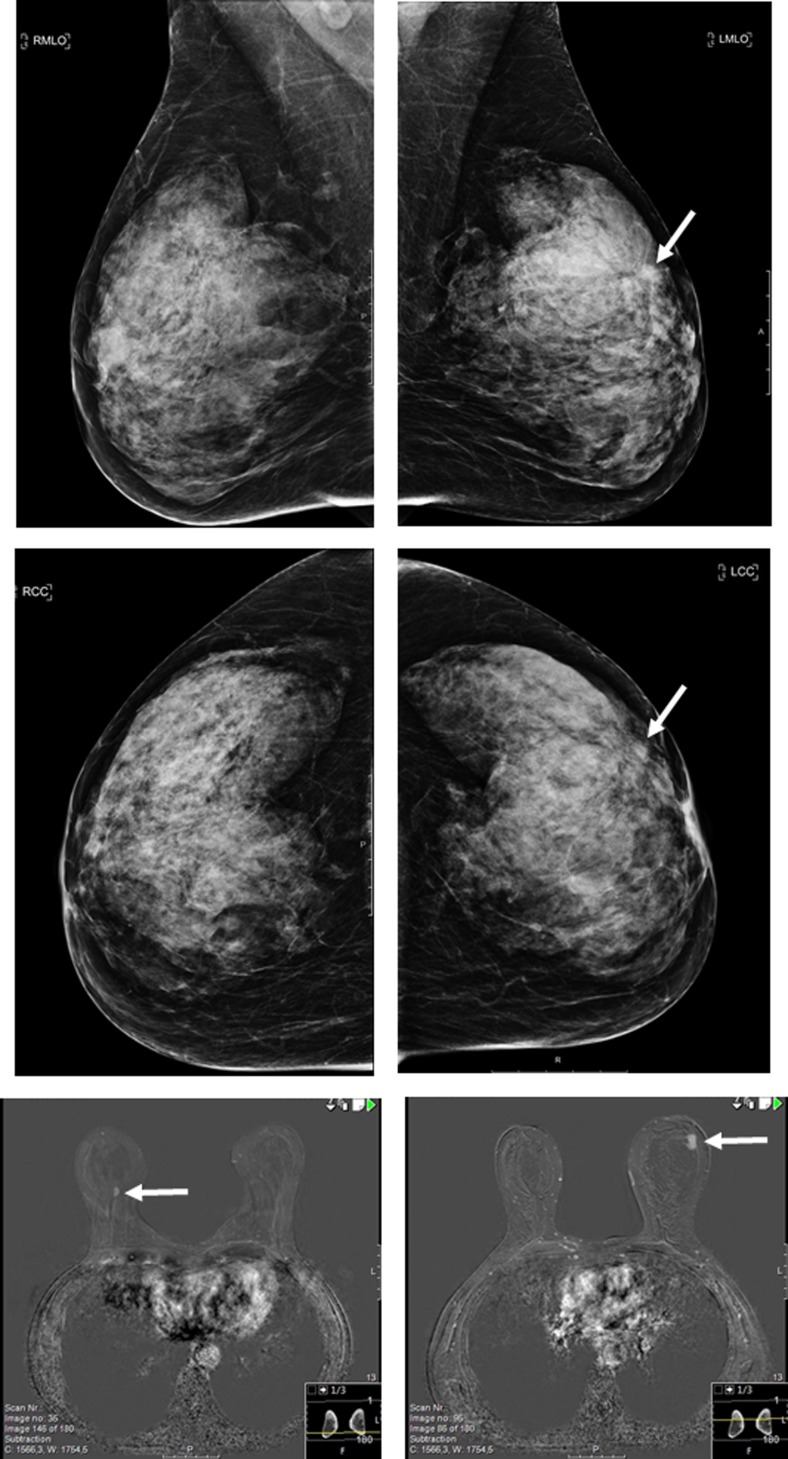
A 64-year-old woman was recalled for a BI-RADS 4 mass in the left breast. Percutaneous core biopsy showed invasive ductal carcinoma and MRI was performed to determine the extent of the disease. MRI demonstrated a BIRADS 4 mass in the right breast, which was occult at subsequent target ultrasound. MRI guided vacuum assisted biopsy revealed invasive lobular carcinoma. Breast conserving surgery yielded and invasive ductal carcinoma of 8 mm in the left breast (sentinel node negative, B&R II, ER+, PR+, Her2-) and an invasive lobular carcinoma of 6 mm in the right breast (sentinel node negative, B&R I, ER+, PR-, Her2-)

Of the 129 lesions detected in the contralateral breast, 20 (15.5%) proved to be malignant (Table 3). The majority of the malignancies presented themselves as a suspicious mass at clinical mammography (eight cases, 47.1%) or breast MRI (seven cases, 41.2%). Of the nine contralateral breast cancers detected at mammography, two were visible only at breast tomosynthesis and not at the full-field digital mammogram. Of the seven cancers visible at the latest screening mammogram in retrospect, respectively four were considered to be missed and three showed a minimal sign. As mentioned previously, the remaining three contralateral cancers presented as palpable breast lesions, not primarily detected at breast imaging. In 16 of the 20 women with a breast cancer diagnosed in the contralateral breast, the mammographic abnormality at screening turned out to be malignant as well, resulting in 16 bilateral cancer cases. In the four remaining cases, the contralateral lesion turned out to be malignant, and the mammographic abnormality at screening turned out to be benign. One of these cases is presented in Fig. 2. The majority of benign lesions detected in the contralateral breast comprised cysts (52.3%, 57/109) and fibroadenomas (13.8%, 15/109).

**Table 3:** Characteristics and outcome of lesions in the contralateral breast, detected at clinical breast imaging following recall

	Final outcome	
Detection method and radiological abnormality, No (%)	Benign	Malignant
Mammography^a^		
Suspicious mass Suspicious calcifications Suspicious mass with calcifications Architectural distortion	81 (65.2) 8 (6.5) 1 (0.8) 2 (1.6)	6 (35.3) 1 (5.9) 0 0
Tomosynthesis-only		
Suspicious mass	9 (7.3)	2 (11.8)
Breast MRI		
Suspicious mass Focal non-mass enhancement	19 (15.3) 4 (3.2)	7 (41.2) 1 (5.9)

^a^With or without breast tomosynthesis

### Comparison of unilateral, screen-detected abnormalities with those detected in the contralateral, non-recalled breast

Of the recalled lesions and contralateral, non-recalled lesions, respectively 22.6% and 15.5% proved to be malignant (*p* = 0.06; Table 4). The type of assessment after recall (clinical breast imaging versus biopsy in addition to clinical breast imaging) was similar for both groups. The positive predictive value of biopsy, however, was higher for recalled lesions (53.1% vs 36.4%, *p* = 0.01). The tumour characteristics and final surgical treatment of unilateral, screen-detected cancers were similar with those of cancers diagnosed in the contralateral, non-recalled breast (Table 5).

**Table 4:** Overall screening outcome of recalled lesions versus lesions analysed in the contralateral, non-recalled breast

	Recalled lesions^a^	Lesions assessed in the contralateral, non-recalled breast	*p* value
	*n* = 3,995	*n* = 129	
Screening outcome, No (%)			0.06
False positive True positive	3,094 (77.4) 901 (22.6)	109 (84.5) 20 (15.5)	
Type of assessment after recall, No (%)			0.98
Clinical breast imaging Clinical breast imaging + biopsy	2,297 (57.5) 1,698 (42.5)	74 (57.4) 55 (42.6)	
Positive predictive value of biopsy, %	53.1	36.4	0.01

^a^Dominant mammographic abnormality in case of multiple recalled lesions

**Table 5:** Tumour characteristics of unilateral, screen-detected cancers versus cancers diagnosed in the contralateral, non-recalled breast

	Unilateral SDCs (*n* = 901)	Contralateral cancers (*n* = 20)	*p* value
Type of cancer, No (%)			0.4
DCIS Invasive	162 (18.0) 739 (82.0)	2 (10.0) 18 (90.0)	
Histology of invasive cancers, No (%)			0.2
Ductal Lobular Ductolobular Other	581 (78.6) 91 (12.3) 23 (3.1) 44 (6.0)	12 (66.7) 5 (27.8) 0 1 (5.6)	
Tumour stage of invasive cancers, No (%)			0.97
T1 T2+ Unknown	569 (77.0) 169 (22.9) 1 (0.1)	14 (77.8) 4 (22.2) 0	
Lymph node status of invasive cancers, No (%)			0.7
N+ N- Unknown	164 (22.2) 561 (76.0) 14 (1.9)	5 (27.8) 13 (72.2) 0	
Bloom & Richardson grade, No (%)			0.8
I II III Unknown	329 (44.5) 318 (43.0) 87 (11.8) 5 (0.7)	8 (44.4) 9 (50.0) 1 (5.6) 0	
Oestrogen receptor status, No (%)			0.4
Positive Negative Unknown	667 (90.3) 69 (9.3) 3 (0.4)	18 (100) 0 0	
Progesterone receptor status, No (%)			0.5
Positive Negative Unknown	520 (70.4) 216 (29.2) 3 (0.4)	15 (83.3) 3 (16.7) 0	
Her2/Neu receptor status, No (%)			0.8
Positive Negative Unknown	72 (9.7) 663 (89.7) 4 (0.5)	1 (5.6) 17 (94.4) 0	
Final surgical treatment, No (%)			0.9
Breast conserving surgery Mastectomy No surgery Unknown	726 (80.6) 163 (18.1) 11 (1.2) 1 (0.1)	17 (85.0) 3 (15.0) 0 0	

*DCIS* ductal carcinoma in situ

## Discussion

In the current study, we found that one out of 30 recalled women underwent analysis of a contralateral, non-recalled breast lesion and about one out of seven of these latter lesions proved to be malignant. The positive predictive value of biopsy of contralateral abnormalities, detected following recall, was lower than that of screen-detected lesions. Contralateral cancers and screen-detected cancers showed similar histological tumour characteristics, tumour stage and surgical treatment.

In retrospect, four out of 20 contralateral malignancies in our series were considered to be missed at the latest screening examination. Setz-Pels et al. [6] found that the sensitivity of screening mammography for the detection of bilateral breast cancer is less than 20%. The authors concluded that both screening radiologists and clinical radiologists should pay vigorous attention to the contralateral breast in order to detect bilateral malignancies without diagnostic delay. However, they do not provide information on the overall presence and work-up of contralateral, non-recalled breast abnormalities.

We found that 3.2% of recalled women underwent assessment of a contralateral, non-recalled breast abnormality. The majority of these lesions were detected at clinical mammography and/or breast tomosynthesis. Tomosynthesis is increasingly used, both at screening mammography and in a diagnostic setting. The addition of tomosynthesis to digital screening mammography may increase the cancer detection rate. A recent US study reported a decreased recall rate 3 years after the introduction of breast tomosynthesis (from 10.4 to 9.0%, *p* < 0.001), as well as an increased positive predictive value of recall (from 4.4 to 6.7%, *p* = 0.02) and a decreased interval cancer rate. A UK study found an increased specificity when adding tomosynthesis to digital screening mammography (70% versus 57%, *p* < 0.001) and an increased sensitivity for cancer detection in dense breasts (93% versus 86%, *p* = 0.03) [9, 21].

Tomosynthesis is not yet implemented in the Dutch screening mammography setting. An increasing number of hospitals have this modality at their disposal. In our series, two of the contralateral breast cancers were visible only at breast tomosynthesis and not at the full-field digital mammogram. It is likely that the intensified use of tomosynthesis in the work-up of recalled women will increase the number of lesions detected in contralateral, non-recalled breasts [7, 8]. A substantial number of contralateral lesions in our study were detected at breast MRI. Again, it can be expected that a further increase in the use of MRI, as well as the introduction of new and promising imaging modalities such as contrast-enhanced spectral mammography [22, 23], will increase the number of breast lesions detected in the contralateral breast of recalled women.

Additional percutaneous biopsy was performed in 43% of these lesions, with a lower positive predictive value of biopsy (36.4%) than the one observed for recalled lesions (53.1%, *p* < 0.01). Excision biopsy, all with benign final pathology, was performed in five women. In some rare cases, excision biopsy may be necessary to establish a final diagnosis. Also, some women may desire excision of a benign lesion. Nevertheless, one should aim to minimise excision biopsy for diagnostic purposes as it has been shown that the sensitivity of screening mammography is lower in women after benign breast surgery [24].

We found no statistically significant differences in the proportions of malignancies among recalled lesions and contralateral, non-recalled malignant lesions (22.6% versus 15.5%). This study could be statistically underpowered to detect a clinically relevant difference due to small patient numbers. The observed trend of a lower cancer risk for contralateral lesions may be due to the fact that screening radiologists have no other tool than the screening mammogram and they have to keep recall percentages within acceptable limits. On the other hand, clinical radiologists are likely to have a lower threshold to exclude malignancy in mammographic lesions for which a woman has not been recalled.

Diagnosis of bilateral breast cancer was established in 16 of 20 women with a contralateral malignant lesions in the non-recalled breast, and four unilateral cancers were diagnosed in a breast for which a woman had not been recalled. A synchronous diagnosis of bilateral breast cancer may have an impact on the choice of final surgical therapy as these women are more inclined to opt for mastectomy over breast-conserving surgery [25]. The survival of patients with bilateral disease is likely to be worse than that of patients with unilateral disease [26, 27].

Studies have demonstrated an improved cancer detection when MRI or whole-breast ultrasound is added to mammography [12, 28, 29]. The increased sensitivity, however, is accompanied by an increase in false-positive findings. Moreover, enhanced use of MRI is correlated with an increase in mastectomy procedures [30–32]. Therefore, the guidelines of the European Society of Breast Imaging and the Dutch guidelines do not promote the standard use of MRI or whole-breast ultrasound in recalled women or in women with pathologically proven breast cancer [16, 17]. Breast ultrasonography should be used mainly as an evaluation tool of lesions detected at mammography or MRI (target ultrasonography or second look) and for the assessment of palpable breast lesions that are occult at mammography. Indications for breast MRI are screening of high risk women, screening of women with dense breasts, detection of additional ipsilateral and contralateral malignancies in invasive lobular cancer, determination of tumour size in dense breasts and in select cases for problem solving of lesions detected at mammography and/or breast ultrasonography.

Recalled women with or without assessment of a non-recalled, contralateral abnormality showed similar baseline characteristics except of a higher mammographic breast parenchyma density in the first group (*p* < 0.001). This may be explained by the fact that additional imaging modalities used in the clinical setting, as mentioned above, have a higher sensitivity for lesion detection compared to mammography, especially in dense breasts [10, 12, 29, 30, 33, 34].

It seems remarkable that three out of 20 detected contralateral malignancies were diagnosed by physical examination only. Contrary to a common perception that imaging obviates the need for physical examination, it remains a mainstay of diagnosis and an integral part of breast awareness and (self-)screening [35].

Tumour characteristics of cancers diagnosed in the contralateral, non-recalled breast were similar to those of unilateral, screen-detected cancers. A majority of cancers in both groups were of the invasive ductal type, stage T1, lymph node negative and grade I or II. A Dutch study found that contralateral breast cancers detected by screening comprised more lobular cancers and showed less nodal involvement than index cancers or unilateral cancers [6].

Our study has certain strengths and limitations. To our knowledge, we are the first to describe the frequency and characteristics of contralateral breast lesions following recall. The study population is large and recalled women were evaluated in multiple hospitals. Our results, however, may not be representative for other regional or nationwide screening mammography programmes as these programmes may show variations in screening interval (1-3 years), reading strategy (single reading versus double reading), percentage of recalled women and availability of higher-end radiological equipment (e.g. tomosynthesis, stereotactic core-needle biopsy, 3-T MRI) at departments of radiology. Furthermore, as mentioned previously, the number of lesions detected in the contralateral breast may increase in the future as new techniques are increasingly implemented in the setting of clinical breast imaging. Ultimately, a majority of the women with contralateral breast lesions only underwent clinical breast imaging, without additional biopsy. BI-RADS 1 and BI-RADS 2 findings did not receive any radiological follow-up, whereas BI-RADS 3 lesions were either biopsied or received their first radiological follow-up at 6 months. As the follow-up period of all recalled women was 1 year, we cannot rule out that a contralateral lesion with benign follow-up may eventually turn out to be malignant.

In summary, reassessment of the complete screening mammogram, including the contralateral breast after recall, is important as contralateral cancers may be detected. We do not advocate a routine evaluation of the contralateral breast with additional imaging procedures following this reassessment, as this strategy will like not be cost-effective. A timely diagnosis of the contralateral breast may be of influence on the choice of surgical therapy of a screen-detected cancer and survival.

## Abbreviations

CNB: Core-needle biopsy
DBT: Digital breast tomosynthesis
FFDM: Full-field digital mammography
FNAC: Fine-needle aspiration cytology
SCNB: Stereotactic core needle biopsy

## References

[1] Sankatsing VDV, van Ravesteyn NT, Heijnsdijk EAM (2017). The effect of population-based mammography screening in Dutch municipalities on breast cancer mortality: 20 years of follow-up. Int J Cancer.

[2] Otto SJ, Fracheboud J, Looman CW (2003). Initiation of population-based mammography screening in Dutch municipalities and effect on breast-cancer mortality: a systematic review. Lancet.

[3] Welch HG, Prorok PC, O'Malley AJ, Kramer BS (2016). Breast-cancer tumor size, overdiagnosis, and mammography screening effectiveness. N Engl J Med.

[4] Pisano ED, Gatsonis C, Hendrick E (2005). Diagnostic performance of digital versus film mammography for breast-cancer screening. N Engl J Med.

[5] Bernardi D, Macaskill P, Pellegrini M (2016). Breast cancer screening with tomosynthesis (3D mammography) with acquired or synthetic 2D mammography compared with 2D mammography alone (STORM-2): a population-based prospective study. Lancet Oncol.

[6] Setz-Pels W, Duijm LE, Groenewoud JH (2011). Detection of bilateral breast cancer at biennial screening mammography in the Netherlands: a population-based study. Radiology.

[7] Waldherr C, Cerny P, Altermatt HJ (2013). Value of one-view breast tomosynthesis versus two-view mammography in diagnostic workup of women with clinical signs and symptoms and in women recalled from screening. AJR Am J Roentgenol.

[8] Lei J, Yang P, Zhang L, Wang Y, Yang K (2014). Diagnostic accuracy of digital breast tomosynthesis versus digital mammography for benign and malignant lesions in breasts: a meta-analysis. Eur Radiol.

[9] Gilbert FJ, Tucker L, Gillan MG (2015). The TOMMY trial: a comparison of TOMosynthesis with digital MammographY in the UK NHS Breast Screening Programme—a multicentre retrospective reading study comparing the diagnostic performance of digital breast tomosynthesis and digital mammography with digital mammography alone. Health Technol Assess.

[10] Lang K, Andersson I, Zackrisson S (2014). Breast cancer detection in digital breast tomosynthesis and digital mammography-a side-by-side review of discrepant cases. Br J Radiol.

[11] El Sharouni MA, Postma EL, Menezes GL (2016). High prevalence of MRI-detected contralateral and ipsilateral malignant findings in patients with invasive ductolobular breast cancer: impact on surgical management. Clin Breast Cancer.

[12] Brennan ME, Houssami N, Lord S (2009). Magnetic resonance imaging screening of the contralateral breast in women with newly diagnosed breast cancer: systematic review and meta-analysis of incremental cancer detection and impact on surgical management. J Clin Oncol.

[13] Wang SY, Long JB, Killelea BK (2016). Preoperative breast magnetic resonance imaging and contralateral breast cancer occurrence among older women with breast cancer. J Clin Oncol.

[14] Duijm LE, Groenewoud JH, Jansen FH, Fracheboud J, van Beek M, de Koning HJ (2004). Mammography screening in the Netherlands: delay in the diagnosis of breast cancer after breast cancer screening. Br J Cancer.

[15] Sickles EA, D’Orsi CJ, Bassett LW et al (2013) ACR BI-RADS® Mammography. In: ACR BI-RADS® Atlas, Breast Imaging Reporting and Data System. American College of Radiology, Reston

[16] NABON (2012) Richtlijn Mammacarcinoom. Available via http://www.oncoline.nl/mammacarcinoom. Accessed 11 July 2017

[17] Mann RM, Balleyguier C, Baltzer PA (2015). Breast MRI: EUSOBI recommendations for women's information. Eur Radiol.

[18] Sobin LH, Gospodarowicz MK, Wittekind C (2009). TNM classification of malignant tumours.

[19] Maes RM, Dronkers DJ, Hendriks JH, Thijssen MA, Nab HW (1997). Do non-specific minimal signs in a biennial mammographic breast cancer screening programme need further diagnostic assessment?. Br J Radiol.

[20] Perry N, Broeders M, de Wolf C, Tornberg S, Holland R, von Karsa L (2008). European guidelines for quality assurance in breast cancer screening and diagnosis. Fourth edition--summary document. Ann Oncol.

[21] McDonald ES, Oustimov A, Weinstein SP, Synnestvedt MB, Schnall M, Conant EF (2016). Effectiveness of digital breast tomosynthesis compared with digital mammography: outcomes analysis from 3 years of breast cancer screening. JAMA Oncol.

[22] Lobbes MB, Lalji U, Houwers J (2014). Contrast-enhanced spectral mammography in patients referred from the breast cancer screening programme. Eur Radiol.

[23] Lalji UC, Houben IP, Prevos R (2016). Contrast-enhanced spectral mammography in recalls from the Dutch breast cancer screening program: validation of results in a large multireader, multicase study. Eur Radiol.

[24] van Breest Smallenburg V, Duijm LE, Voogd AC (2012). Lower sensitivity of screening mammography after previous benign breast surgery. Int J Cancer.

[25] O'Brien JA, Ho A, Wright GP (2015). Breast-conserving surgery in bilateral breast cancer. Ann Surg Oncol.

[26] Heron DE, Komarnicky LT, Hyslop T, Schwartz GF, Mansfield CM (2000). Bilateral breast carcinoma: risk factors and outcomes for patients with synchronous and metachronous disease. Cancer.

[27] Schaapveld M, Visser O, Louwman WJ (2008). The impact of adjuvant therapy on contralateral breast cancer risk and the prognostic significance of contralateral breast cancer: a population based study in the Netherlands. Breast Cancer Res Treat.

[28] Iacconi C, Galman L, Zheng J (2016). Multicentric cancer detected at breast MR imaging and not at mammography: important or not?. Radiology.

[29] Melnikow J, Fenton JJ, Whitlock EP (2016). Supplemental screening for breast cancer in women with dense breasts: a systematic review for the U.S. Preventive Services Task Force. Ann Intern Med.

[30] Houssami N, Abraham LA, Onega T (2014). Accuracy of screening mammography in women with a history of lobular carcinoma in situ or atypical hyperplasia of the breast. Breast Cancer Res Treat.

[31] Chandwani S, George PA, Azu M (2014). Role of preoperative magnetic resonance imaging in the surgical management of early-stage breast cancer. Ann Surg Oncol.

[32] Vriens IJH, Keymeulen K, Lobbes MBI (2017). Breast magnetic resonance imaging use in patients undergoing neoadjuvant chemotherapy is associated with less mastectomies in large ductal cancers but not in lobular cancers. Eur J Cancer.

[33] Sprague BL, Stout NK, Schechter C (2015). Benefits, harms, and cost-effectiveness of supplemental ultrasonography screening for women with dense breasts. Ann Intern Med.

[34] Lee CI, Cevik M, Alagoz O (2015). Comparative effectiveness of combined digital mammography and tomosynthesis screening for women with dense breasts. Radiology.

[35] Provencher L, Hogue JC, Desbiens C (2016). Is clinical breast examination important for breast cancer detection?. Curr Oncol.

