# Correction to: Frequency and characteristics of contralateral breast abnormalities following recall at screening mammography

**DOI:** 10.1007/s00330-018-5532-x

**Published:** 2018-06-25

**Authors:** Joost R. C. Lameijer, Angela MP Coolen, Adri C. Voogd, Luc J. Strobbe, Marieke W. J. Louwman, Dick Venderink, Vivian C. Tjan-Heijnen, Lucien E. M. Duijm

**Affiliations:** 1 Department of Radiology, Catharina Hospital, Michelangelolaan 2, 5623EJ, Eindhoven, The Netherlands; 2 Department of Radiology, Elisabeth-Tweesteden Hospital (ETZ), Hilvarenbeekseweg 60, 5022 GC Tilburg, The Netherlands; 3 Department of Epidemiology, Maastricht University, P Debyelaan 1, 6229 HA Maastricht, The Netherlands; 4 Department of Research, Netherlands Comprehensive Cancer Organization (IKNL), PO Box 19079, 3501 DB Utrecht, The Netherlands; 5 Department of Internal Medicine, Division of Medical Oncology, GROW Maastricht University Medical Centre, PO Box 5800, 6202 AZ Maastricht, The Netherlands; 6 Department of Radiology, Canisius Wilhelmina Hospital, Weg door Jonkerbos 100, 6532 SZ Nijmegen, The Netherlands; 7 Dutch Expert Centre for Screening, PO Box 6873, 6503 GJ Nijmegen, The Netherlands

**Correction to: Eur Radiol**

10.1007/s00330-018-5370-x

The original version of this article, published on 17 April 2018, unfortunately contained a mistake. The following correction has therefore been made in the original:

The name of Angela MP Coolen and the affiliations were presented incorrectly. The corrected author list is given above; the corrected affiliations are given below. The original article has been corrected.

